# 

**Published:** 1922-03

**Authors:** 


### HOSPITAL and HEALTH REVIEW

Established 1886 No. 1848. Vol. LXXI.

New Series. Vol. 1. No. 6 March 1922 Price Sixpence

CONTENTS.

| | PAGE |
|---|---|
| Notes and Comments | 151 |
| The Fundamental Biological Factors Underlying the Incidence of Tuberculosis.—II. R. J. Ewart, M.D., D.Sc., F.R.C.S. | 157 |
| Medical Education and the Public Health | 159 |
| Hospital Planning in America | 161 |
| Health with Economy: The Position on the South Coast.—I. Eastbourne | 163 |
| Hospital Officials' Pensions | 164 |
| Medicine in Ancient Egypt and Ethiopia M. B. Ray, D.S.O., M.D. | 165 |
| Tobacco and the Worker | 167 |
| Reviews | 168 |
| Passing Events and Topics | 171 |
| News in Brief | 174 |
| Parliamentary Questions and Answers | 176 |
| Prize Leaflets | 177 |

